# Pulmonary Artery Cement Embolism after a Vertebroplasty

**DOI:** 10.1155/2015/582769

**Published:** 2015-06-28

**Authors:** Anas Nooh, Fahad H. Abduljabbar, Ahmed H. Abduljabbar, Peter Jarzem

**Affiliations:** ^1^McGill Scoliosis & Spine Centre, McGill University Health Centre, Montreal, QC, Canada H3A 0G4; ^2^Department of Orthopedic Surgery, King Abdulaziz University, Jeddah, Saudi Arabia; ^3^Department of Diagnostic Radiology, Dalhousie University, Halifax, NS, Canada B3H 4R2

## Abstract

*Background Context*. Vertebroplasty is a minimally invasive procedure most commonly used for the treatment of vertebral compression fractures. Although it is relatively safe, complications have been reported over time. Among those complications, massive cement pulmonary embolism is considered a rare complication. Here we report a case of massive diffuse cement pulmonary embolism following percutaneous vertebroplasty for a vertebral compression fracture. *Study Design*. Case report. *Methods*. This is a 70-year-old female who underwent vertebroplasty for T11 and T12 vertebral compression fracture. *Results*. CT-scan revealed an incidental finding of cement embolism in the pulmonary trunk and both pulmonary arteries. Since the patient was asymptomatic, she was monitored closely and she did not need any intervention. *Conclusion*. Vertebroplasty is a minimally invasive procedure used for treatment of vertebral compression fracture. Despite the low rate of complications, a pulmonary cement embolism can occur. The consequences of cement embolism range widely from being asymptomatic to embolism that can cause paralysis, radiculopathy, or a fatal pulmonary embolism.

## 1. Introduction

Vertebroplasty was first introduced in 1987 by Galibert et al. [1]. It is a minimally invasive procedure that is most commonly used as a treatment for painful vertebral compression fractures and painful bone metastases. In this procedure, polymethylmethacrylate (PMMA) is injected directly into the vertebral body through its pedicle, to restore the height partially, stabilize bony trabeculae, and alleviate pain. Although it is a relatively safe procedure, complications have been reported [2]. Among those complications, cement pulmonary embolism can occur as a sequel of extravertebral leakage of cement into the venous system [3]. Pulmonary cement emboli may be encountered in 25% of patients undergoing vertebroplasty, fortunately clinically silent in the vast majority of cases [4]. The consequences of cement embolism range widely from being asymptomatic to embolism that can cause paralysis, radiculopathy, or a fatal pulmonary embolism [5]. Here we report an incidental finding of a cement pulmonary embolism following vertebroplasty for a thoracic compression fracture.

## 2. Case Report

**Figure 1:**
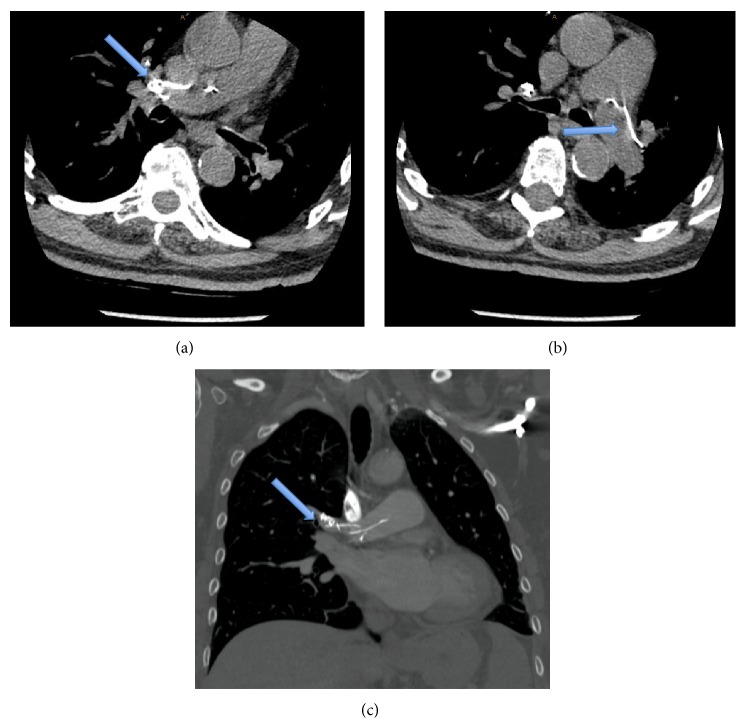
(a) An axial cut of the chest CT-scan of a nonenhanced CT chest showing multiple linear and extremely dense bone cement emboli involving pulmonary trunk and right pulmonary artery. (b) An axial cut of a nonenhanced CT chest showing a cement embolus involving the left main pulmonary artery. (c) A coronal cut of CT pulmonary angiogram also showing the cement emboli at the pulmonary trunk and right main pulmonary artery.

This is a 70-year-old female who is a known case of diabetes mellitus, hypertension, and coronary artery disease. She presented to our clinic in February 2013 with debilitating back pain secondary to degenerative lumbar scoliosis with a multilevel lumbar spinal stenosis. She had posterior spinal fusion from T12 to pelvis with a decompression of the stenotic levels. Two months following her surgery, she presented to our clinic complaining of disabling back pain after a history of falling on her back. This pain was exacerbated by activity and relieved by rest. However, she had no change in her neurological status. Clinical and radiographic findings confirmed T11 and T12 compression fractures. As the patient was symptomatic, the decision has been made to proceed with vertebroplasty and in this way avoid a more extensive surgery with rods and screws. Following general anesthesia, she was positioned in a prone position. Then, under direct fluoroscopy, the correct levels were confirmed followed by a standard percutaneous insertion of trocars into T11-T12 pedicles. High viscosity bone cement was injected into T11 and T12 vertebral bodies under fluoroscopic guidance. At T12, the bilateral trocars were placed using an in-out in technique aiming the tip of the trocar just under the pedicle screws at T12. A bipedicular approach was also used at T11 to inject bone cement. High viscosity bone cement was injected into T11 and T12 vertebral bodies under fluoroscopic guidance. The total volume of injected cement was 4 cc in each level. The patient tolerated the procedure and was discharged uneventfully. Six months following her vertebroplasty, she presented to our clinic with a history of trauma causing back pain, diminishing her ability to ambulate, and worsening neurological symptoms. On clinical examination, she had tenderness over the thoracolumbar spine and generalized weakness of the lower extremity. Radiographs demonstrated a significant 45-degree junctional kyphosis at T11-T12 with a loss of sagittal balance. Subsequent CT-scan showed an incidental finding of a cement pulmonary embolism in the pulmonary trunk and right and left main pulmonary arterial systems (Figure 1). Patient's spine sagittal imbalance was treated surgically by extending her spinal fusion up to T2 to restore her sagittal alignment. Nine months following her vertebroplasty, the patient started to have a new onset of shortness of breath. Pulmonary embolism was the main working diagnosis. For this reason, a complete cardiorespiratory work-up was carried out. CT pulmonary angiogram demonstrated an unchanged cement embolus within the pulmonary trunk extending to both main pulmonary arteries. Echocardiography revealed an ejection fraction of 60% and a pulmonary artery systolic pressure of 35 mmHg, which is considered normal. Pulmonary function testing was normal. Dobutamine cardiac echocardiography was also unremarkable. There was no physiological explanation for her shortness of breath, and her cement embolism is just an incidental finding. Moreover, her shortness of breath was mainly after exertion and responds very well to nitroglycerine. Because there is no interval change in the patient's pulmonary or cardiac function tests, the respirology and cardiology consultants recommended close monitoring for any signs and symptoms suggestive of worsening embolism. Serial cardiac and pulmonary assessments will be carried out looking for increased pulmonary artery pressure as an indicator for the removal of the cement embolus with the aid of an interventional radiologist.

## 3. Discussion

In this case report, we presented an incidental finding of a cement embolism in the pulmonary trunk and both pulmonary arteries following vertebroplasty to emphasize the importance of close monitoring of patients following this procedure. Around 100 cases of cement embolism following vertebroplasty were reported in the literature with the majority being asymptomatic [6]. Previous studies reported cement extrusion into the venous system during the procedure as the primary cause of cement embolisms [7]. It has been shown that multiple factors can contribute to cement extrusion starting with the volume of injected cement. The volume of cement injected is directly related to cement leakage complications [8]. The current recommendation is to inject less than 5 mL per vertebral body [9]. In addition, some studies showed that cement viscosity has also been identified to be an important factor in decreasing cement leakage, implying that high viscosity cement can reduce cement leakage [10, 11]. Another factor that is closely related to cement extrusion is the number of pedicles accessed. Bilateral transpedicular approach is preferred to a unilateral approach as it avoids the central part of the disc where the leakages are more likely to occur [12]. Furthermore, cement injection pressure can contribute to cement leakage [12].

Clinical presentation of cement pulmonary embolism varies widely from an incidental radiographic finding to a life-threatening condition. Symptoms can include dyspnea, tachypnea, tachycardia, cyanosis, chest pain, cough, hemoptysis, dizziness, or sweating [13]. Consequently, cement pulmonary embolism may cause sustained increase in the pulmonary artery pressure and thus will lead to a cardiopulmonary failure [14]. In addition, it may activate the inflammatory pathway, which increases the vascular permeability causing adult respiratory distress syndrome (ARDS) [15]. Other systematic manifestations may occur as bone cement can embolize to the heart [16], kidney [17], or brain [18].

While there are no clear guidelines to screen for asymptomatic pulmonary embolisms, some authors recommend routine chest radiographs following vertebroplasty or kyphoplasty in asymptomatic patients [8, 19]. A high-density opacity in a tubular branching pattern, corresponding to pulmonary arterial distribution, is usually suggestive of cement pulmonary embolism [6].

In symptomatic patients, a history of previous kyphoplasty or vertebroplasty and chest radiographs can help in the diagnosis. CT angiogram of the chest remains the gold standard test; nevertheless, complete evaluation of cardiac chambers is necessary as cement may deposit in the right side of the heart, which may cause a sustained increase in the pulmonary artery pressure leading to a subsequent cardiopulmonary failure [16].

Although multiple cases of cement embolism have been reported in the literature, there is no clear treatment strategy for cement pulmonary embolism. The treatment is mainly based on the type of the pulmonary emboli whether it is central or peripheral. A central embolism can involve the main pulmonary trunk and/or the right or left main pulmonary arteries. Any embolism beyond that is considered a peripheral pulmonary embolism.

In our case report, the patient had a central pulmonary embolism, which is asymptomatic.

Treatment options include close observation and clinical revaluation for patients with embolism in the peripheral pulmonary vasculatures. For patients with a central embolism, there is no clear consensus regarding its treatment. Some authors recommended no treatment but monitoring those patients as long as they remain asymptomatic and there are no interval changes clinically or radiographically [9], while other authors recommended that, whether symptomatic or not, they should be treated with anticoagulation therapy for 6 months [19]. Surgical or percutaneous embolectomy was suggested by few authors in case of massive central embolism [6, 20].

Given the number of reported cases of cement embolism following vertebroplasty, we recommend monitoring patients following vertebroplasty closely for symptoms suggestive of cement pulmonary embolism and performing a complete diagnostic work-up including a chest X-ray, CT-scan and an echocardiogram in symptomatic patients.

## 4. Conclusion

Vertebroplasty is an efficient and cost-effective procedure to treat symptomatic vertebral compression fracture. Pulmonary cement embolism is one of the major complications secondary to this procedure; however, most of the time it is asymptomatic. We recommend close monitoring for patients following vertebroplasty. For symptomatic patients, a complete cardiorespiratory work-up is mandatory. Asymptomatic patients can be observed closely for any signs of deterioration either clinically or radiographically. Symptomatic patient can be treated with an anticoagulant for 6 months unless there is a life-threatening condition in which case surgical/radiologic embolectomy is required.
